# Diabetic Muscle Infarction: Resolution of Rare Microangiopathy with Over-The-Counter Medication

**DOI:** 10.1155/2021/5555051

**Published:** 2021-07-09

**Authors:** Roopam Jariwal, Nadia Raza, Catherine Cadang, Syed Rahman, David Contreras, Ramanjeet Sidhu, Janpreet Bhandohal

**Affiliations:** ^1^UCLA-Kern Medical Center, 1700 Mount Vernon Avenue, Bakersfield, CA 93306, USA; ^2^American University School of Medicine, 1 University Drive at, Jordan Dr, Cupecoy, Netherlands

## Abstract

Diabetic muscle infarction (DMI) is a rare complication of poorly controlled type 1 and type 2 diabetes seen mostly in those who have already experienced microvascular complications. Currently, the incidence and prevalence of DMI are difficult to conclude, and there is no clear algorithm or standard of care in managing this condition. Pathogenesis of the microangiopathy of DMI remains unclear. A major finding in this investigation of DMI emphasizes that, within 2–17 weeks, patients who initiate low-dose acetylsalicylic acid, bed rest, and close outpatient follow-up see significant size reduction of lower extremity mass and complete resolution of pain without being subjected to invasive muscle biopsy.

## 1. Introduction

Diabetic Muscle Infarction (DMI), also referred to as Diabetic Myonecrosis (DM), is a rare complication of poorly controlled type 1 and type 2 diabetes affecting the thigh muscles in 80% of cases and lower leg muscles in 17% of cases [1]. DMI can mimic other conditions such as cellulitis, deep vein thrombosis, or even necrotizing fasciitis leading to misdiagnosis and inappropriate management due to scarce literature [2]. It is usually seen in those with microvascular complications such as retinopathy, neuropathy, and most commonly, nephropathy. The incidence and prevalence of DMI are difficult to conclude. In a 2006 systematic review, 115 cases were reported, but the actual incidence of diabetic muscle infarction is speculated to be much higher with a predilection for those who are middle aged and of female sex [3]. A systematic review published in 2015 reported 170 cases of DMI since its first introduction to the literature in 1965. This included 126 initial presentations and 44 episodes of recurrence, with a mean age of presentation being 45 years and a range of 20 to 67 years [4]. There is still no clear algorithm or standard of care in managing this condition. In this case report, we explore both presenting signs and efficient management of DMI in an effort to prevent subjecting patients to invasive and unnecessary procedures such as biopsies and surgical debridement that ultimately may lead to significant morbidity.

## 2. Case Presentation

**Figure 1:**
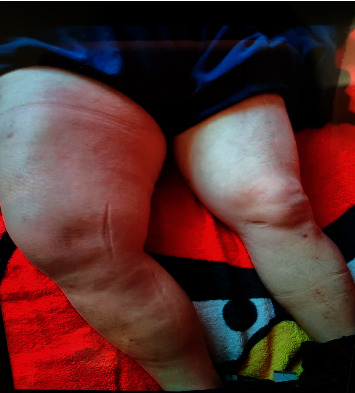
Physical exam showing significant edema of the right thigh.

A 52-year-old Hispanic male with newly diagnosed diabetes mellitus, hypertension, and ischemic cardiomyopathy presented with a one-month history of nonradiating right thigh erythema, pain, and swelling. He worked as a warehouse clerk; however, due to the severe, debilitating pain to his right thigh, the patient was on disability. He required maximal assistance in ambulation and, at times, even a wheelchair. He denied a history of fever, physical trauma, or intravenous drug use. The patient reported several emergency department and urgent care visits prior to our evaluation with no significant improvement on oral antibiotics for presumed cellulitis. Vital signs on presentation included blood pressure 108/69 mm Hg, heart rate 77 beats per minute, temperature 37.5°C, respiratory rate 18, and 96% on room air. On physical examination, a hard, tender, erythematous 7 × 4 inch right anterior thigh mass with accompanying right upper thigh edema was noted (as shown in Figure 1). Distal pulses were 2+ on palpation. Right lower extremity had 3+ pitting edema, while the left lower extremity had 2+ pitting edema. Severe tenderness was also noted in the right groin region with associated painful groin lymphadenopathy.

**Figure 2:**
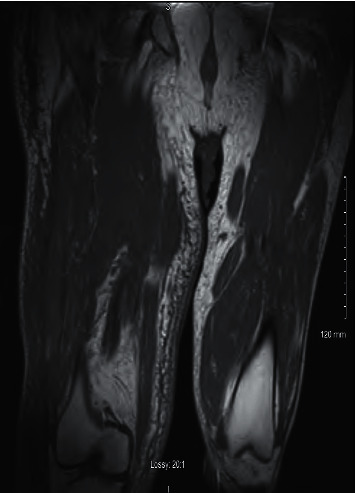
MRI without contrast of the right femur: bilateral inflammatory changes manifested by extensive subcutaneous edema with interfascial free fluid as well as severe myositis. Disproportionate enlargement of the right vastus medialis muscle which is diffusely heterogeneous with a patchy area of intramuscular hemorrhage is shown.

White blood cell count was 10.7 × 10^3 cells/mcl; other laboratory tests did show signs of systemic inflammation including elevated erythrocyte sedimentation rate 49 mm and elevated C-reactive protein 7.98 mg/dL. Aldose value was 3.8 units/L. The patient had an elevated creatinine 1.31 mg/dL, blood urea nitrogen 25 mg/dL, urine albumin/creatinine ratio 1,342.8 mcg/mg Cr, and creatinine kinase 844 unit/L, hemoglobin A1c (HbA1c) was 10.3%, and blood cultures were negative. Laboratory values to diagnose polymyositis and dermatomyositis were negative. A venous Doppler ultrasound of the right leg was negative for deep vein thrombosis. Magnetic resonance imaging (MRI) without contrast of the femur demonstrated bilateral inflammatory changes with disproportionate enlargement and diffuse heterogeneity of the right vastus medialis muscle with patchy areas of intramuscular hemorrhage, extensive subcutaneous edema, interfascial free fluid, and severe myositis (as shown in Figure 2).

Due to lack of improvement of symptoms on antibiotics and poorly controlled diabetes, the diagnosis of diabetic muscle infarction was considered. The treatment consisted of analgesia, low-dose aspirin, bed rest, and close outpatient follow-up. Upon discharge from the hospital, the patient was referred to a tertiary center for muscle biopsy; however, this was delayed due to the coronavirus disease 2019 (COVID-19) pandemic. Three months after being discharged from the hospital, the patient endorsed a significant size reduction of right thigh mass, complete resolution of pain, and endorsed being able to ambulate without assistance. With time, rest, and excellent adherence to low-dose aspirin, he no longer required a biopsy due to complete resolution of symptoms.

## 3. Discussion

Diabetic muscle infarction (DMI) is a rare, often-missed diagnosis that causes significant pain and disability for patients. The mean duration of symptoms before presenting for care is about four weeks, and the time to resolution ranges from 2 to 17 weeks with an average of four weeks [5]. As discussed by Smith et al., typical clinical presentation of DMI includes abrupt onset of pain in the affected muscle accompanied by local swelling with subsequent partial resolution and appearance of a palpable painful mass. DMI most frequently affects the thigh, and the quadriceps was the most commonly affected muscle; calf involvement has also been reported. It is mostly unilateral; however; bilateral lower extremity involvement has been reported. The muscles more frequently affected were the vastus lateralis and the vastus medialis, as seen in our patient's case with Figure 2 showing significant inflammatory changes of the right vastus medialis muscle. The characteristic feature of DMI in MRI is an increased signal from the affected muscle area (intramuscular and perimuscular tissues) in *T*2-weighted, inversion-recovery, and gadolinium-enhanced images and isointense or hypointense areas on *T*1-weighted images, secondary to increased water content from edema and inflammatory changes that accompany the infarction [6]. DMI may be diagnosed by means of a combination of clinical presentation and radiological imaging. The most valuable diagnostic technique is MRI; axial images are the best plane for diagnosis, although coronal and sagittal images may be useful to document the extent of involvement [7]. Muscle biopsy is not recommended, but typically shows muscle fiber necrosis, inflammatory cell infiltrates, and hyalinization of the blood vessels with luminal narrowing [8].

The pathogenesis of DMI remains to be clarified. The most likely hypothesis is that muscle infarction is caused by vascular disease such as arteriosclerosis and diabetic microangiopathy. Epidemiologic data suggest that the presentation of acute or insidious lower limb pain may be linked to arteriosclerotic factors such as endothelial and platelet dysfunction [9]. Some literature suggests an alteration in the coagulation-fibrinolysis system as the cause of DMI which is supported in a case series by Palmer and Greco who reported two patients with DMI and antiphospholipid syndrome [10]. Although it is difficult to assess the relative contribution of microvascular complications of diabetes versus antiphospholipid antibodies to the DMI, the experiences of Palmer and Greco were further supported by Gargiulo et al. that indicate the antiphospholipid antibodies as contributing factors in the progression of diabetes complications, acting as a link between the immunological and hemostatic systems in the pathogenesis of diabetic microangiopathy. Our patient tested negative for antiphospholipid antibodies.

Treatment with low-dose aspirin decreases mean recovery time by 2.5 weeks through its antiplatelet and anti-inflammatory process [11]. Aspirin irreversibly blocks prostaglandin *H* synthase (cyclooxygenase-1) in both platelets and megakaryocytes, preventing the formation of thromboxane *A*2, a potent vasoconstrictor and platelet aggregant [12]. This further supports its use in slowing the microischemic complications seen in poorly controlled diabetes mellitus. The delay in muscle biopsy due to the COVID-19 pandemic was a blessing in disguise for our patient as it gave him the time to heal with over-the-counter low-dose aspirin. An astute physician will carry a high index of suspicion for this underdiagnosed condition when evaluating thigh pain and swelling in patients with poorly controlled blood glucose levels. Patients who see significant improvement after the initiation of appropriate glycemic control, rest, and low-dose cetylsalicylic acid within 2–17 weeks should not be subjected to invasive muscle biopsies.
